# Supplementary material for: On the classification of simple and complex biological images using Krawtchouk moments and Generalized pseudo-Zernike moments: a case study with fly wing images and breast cancer mammograms
Source: PeerJ Comput Sci. 2021 Sep 9;7:e698. doi: 10.7717/peerj-cs.698 (PMC8444072; doi:10.7717/peerj-cs.698)
Supplement: Supplemental Information 1 [file peerj-cs-07-698-s001.pdf]

Supplemental File 1 to Classification of simple and complex biological images using Krawtchouk moments and Generalized Pseudo-Zernike moments: a case study with fly wing images and breast cancer mammograms

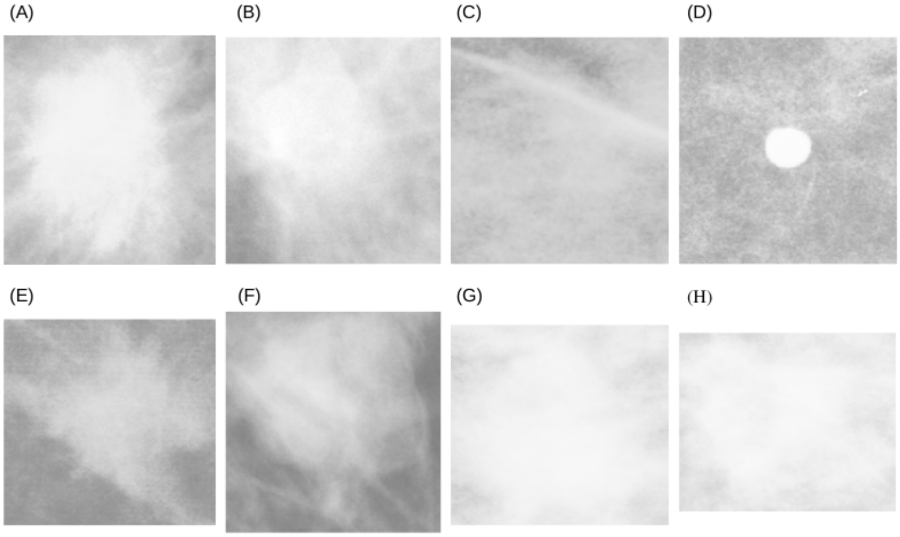

Figure 1: Images used to select the moment order. Benign cases: (a) 344 pixel × 319 pixel; (c) 186 pixel × 178 pixel; (e) 185 pixel × 177 pixel; and (g) 297 pixel × 289 pixel. Malignant cases: (b) 291 × 175 pixel; (d) 185 pixel × 177 pixel; (f) 273 × 282 pixel; and (h) 225 pixel × 273 pixel

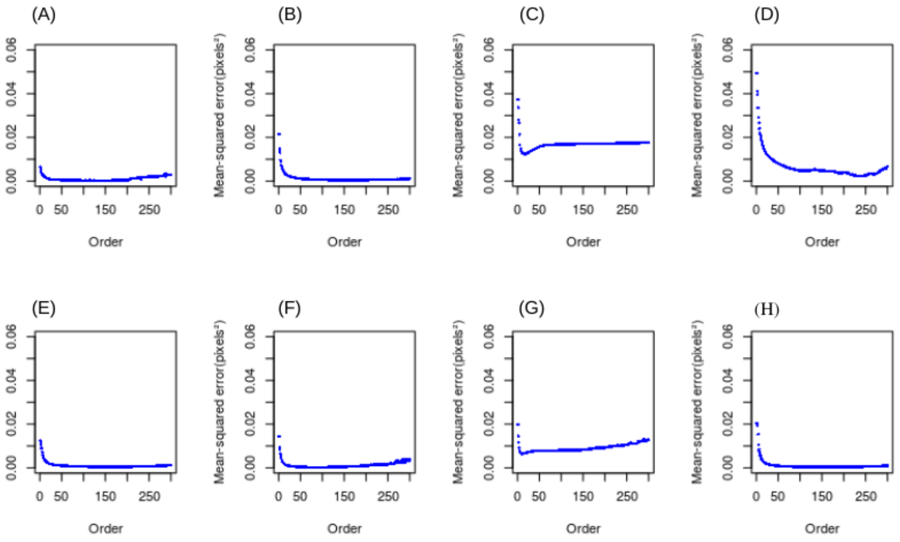

Figure 2: MSE of reconstructed image using KM. (A)-(H) are the MSE of reconstructed images according to the images from 1

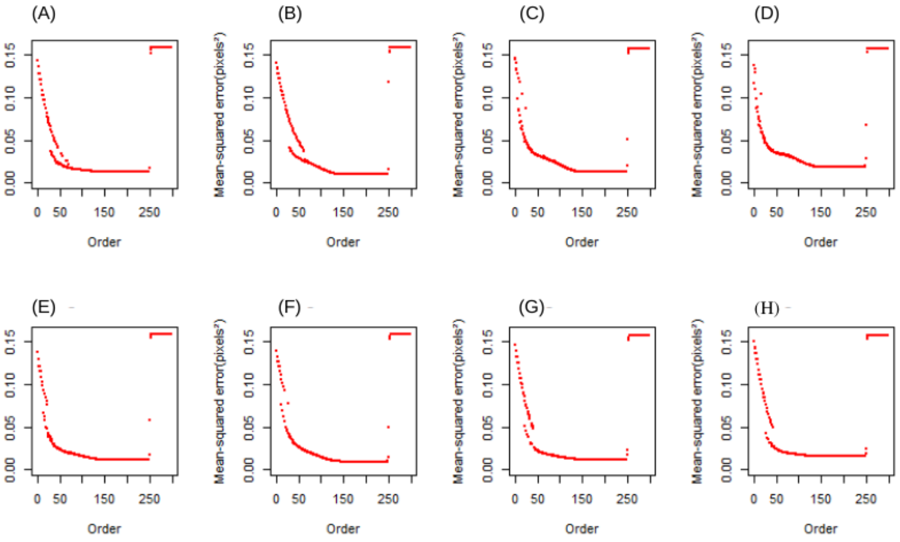

Figure 3: MSE of reconstructed image using GPZM with $\alpha = 0$. (A)-(H) are the MSE of reconstructed images according to the images from 1
