# Supplementary material for: On the classification of simple and complex biological images using Krawtchouk moments and Generalized pseudo-Zernike moments: a case study with fly wing images and breast cancer mammograms
Source: PeerJ Comput Sci. 2021 Sep 9;7:e698. doi: 10.7717/peerj-cs.698 (PMC8444072; doi:10.7717/peerj-cs.698)
Supplement: Supplemental Information 2 [file peerj-cs-07-698-s002.pdf]

Supplemental File 2 to Classification of simple and complex biological images using Krawtchouk moments and Generalized Pseudo-Zernike moments: a case study with fly wing images and breast cancer mammograms

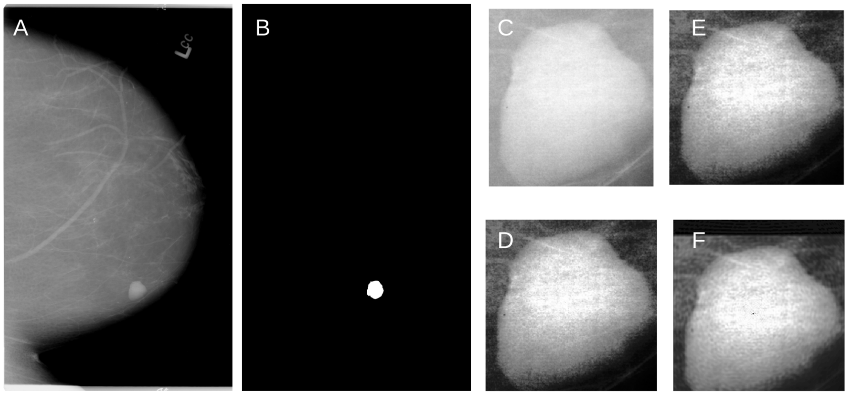

Figure 1: (A) An example of the raw mammogram image showing benign circumscribed breast tumor (index no. P_01712). (B) Binarized mammogram image showing the tumor (white). (C) Region of interest centered on the tumor (296 pixel × 319 pixel) before (D) Enhancement. (E) Reconstruction using KM; (F) Reconstruction using GPZM.

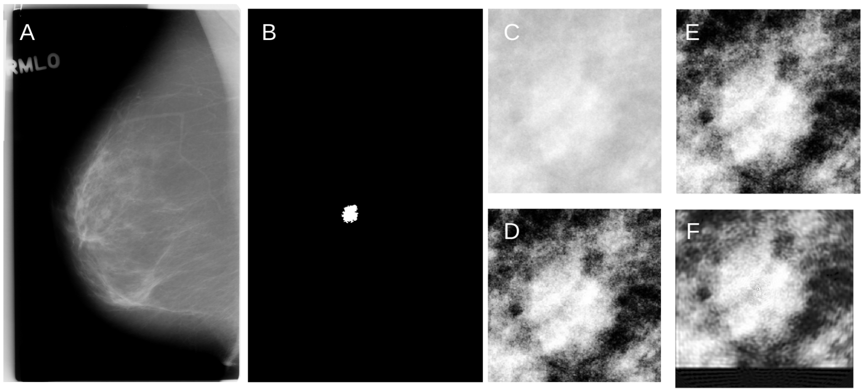

Figure 2: (A) An example of the raw mammogram image showing benign circumscribed breast tumor (index no. P_01395). (B) Binarized mammogram image showing the tumor (white). (C) Region of interest centered on the tumor (259 pixel × 275 pixel) before (D) Enhancement. (E) Reconstruction using KM; (F) Reconstruction using GPZM.

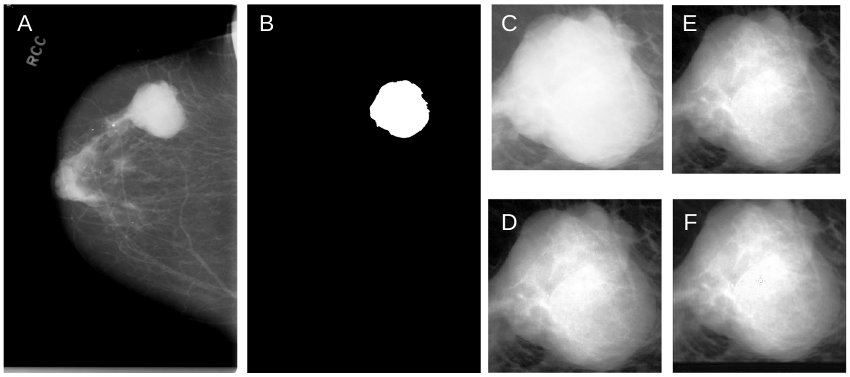

Figure 3: (A) An example of the raw mammogram image showing malignant circumscribed breast tumor (index no. P_01270). (B) Binarized mammogram image showing the tumor (white). (C) Region of interest centered on the tumor (996 pixel × 960 pixel) before (D) Enhancement. (E) Reconstruction using KM; (F) Reconstructed using GPZM.
