# Supplementary figures and images for: On the classification of simple and complex biological images using Krawtchouk moments and Generalized pseudo-Zernike moments: a case study with fly wing images and breast cancer mammograms
Source: PeerJ Comput Sci. 2021 Sep 9;7:e698. doi: 10.7717/peerj-cs.698 (PMC8444072; doi:10.7717/peerj-cs.698)

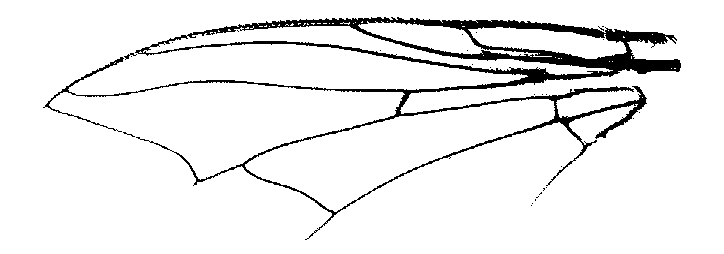

Supplement: Supplemental Information 4 [file peerj-cs-07-698-s004.zip › Supplemental File - Problem 1 Code and Raw Data/binary_images/B.javanica.1.M.png]

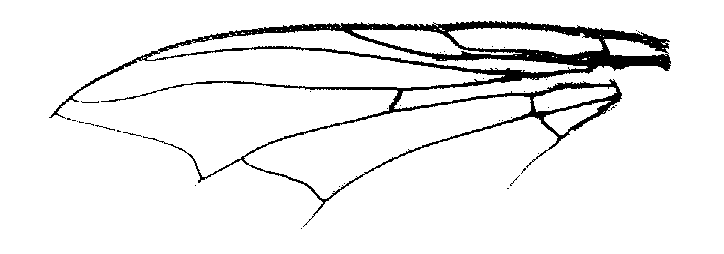

Supplement: Supplemental Information 4 [file peerj-cs-07-698-s004.zip › Supplemental File - Problem 1 Code and Raw Data/binary_images/B.javanica.2.M.png]

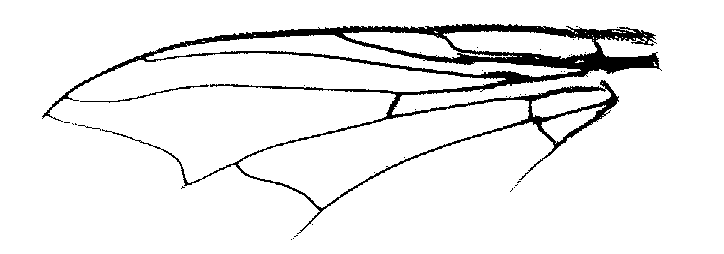

Supplement: Supplemental Information 4 [file peerj-cs-07-698-s004.zip › Supplemental File - Problem 1 Code and Raw Data/binary_images/B.javanica.3.M.png]

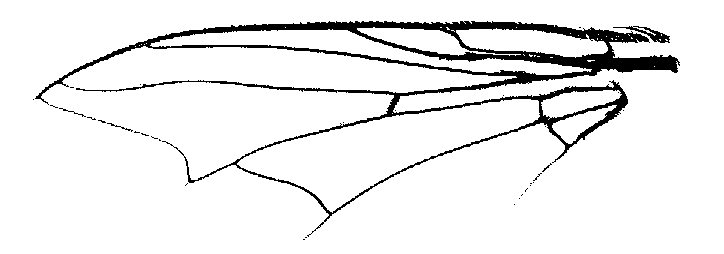

Supplement: Supplemental Information 4 [file peerj-cs-07-698-s004.zip › Supplemental File - Problem 1 Code and Raw Data/binary_images/B.javanica.4.M.png]

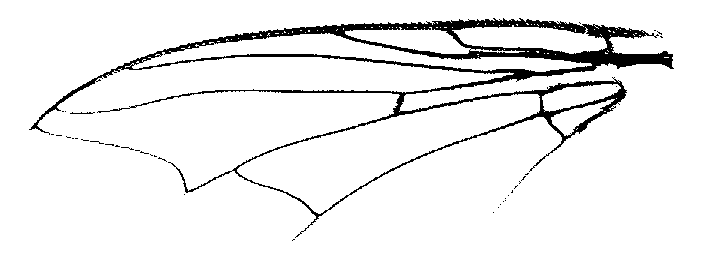

Supplement: Supplemental Information 4 [file peerj-cs-07-698-s004.zip › Supplemental File - Problem 1 Code and Raw Data/binary_images/B.javanica.5.M.png]

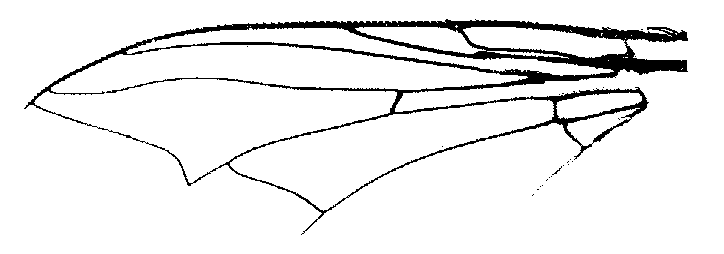

Supplement: Supplemental Information 4 [file peerj-cs-07-698-s004.zip › Supplemental File - Problem 1 Code and Raw Data/binary_images/B.karnyi.1.M.png]

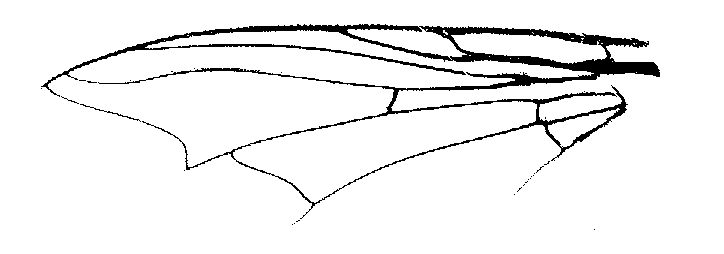

Supplement: Supplemental Information 4 [file peerj-cs-07-698-s004.zip › Supplemental File - Problem 1 Code and Raw Data/binary_images/B.karnyi.2.M.png]

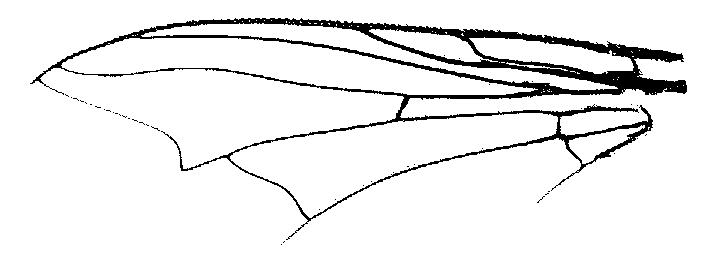

Supplement: Supplemental Information 4 [file peerj-cs-07-698-s004.zip › Supplemental File - Problem 1 Code and Raw Data/binary_images/B.karnyi.3.M.png]

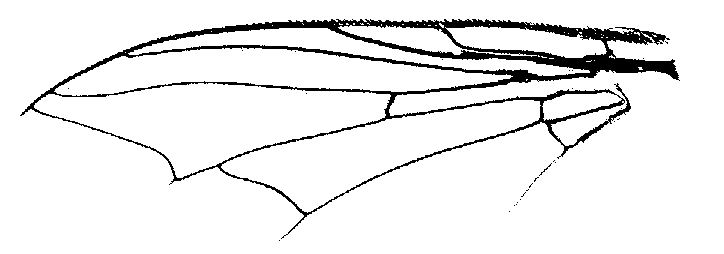

Supplement: Supplemental Information 4 [file peerj-cs-07-698-s004.zip › Supplemental File - Problem 1 Code and Raw Data/binary_images/B.karnyi.4.M.png]

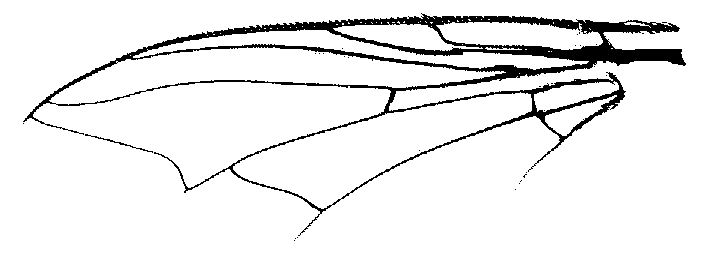

Supplement: Supplemental Information 4 [file peerj-cs-07-698-s004.zip › Supplemental File - Problem 1 Code and Raw Data/binary_images/B.karnyi.5.M.png]

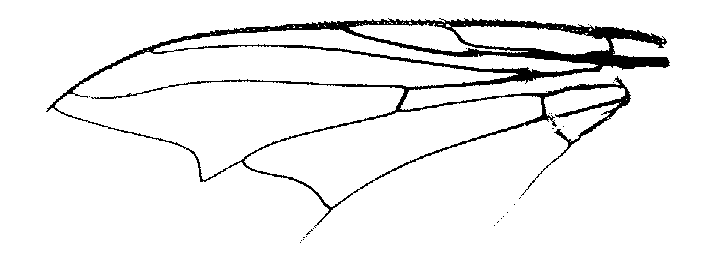

Supplement: Supplemental Information 4 [file peerj-cs-07-698-s004.zip › Supplemental File - Problem 1 Code and Raw Data/binary_images/B.peregrina.1.M.png]

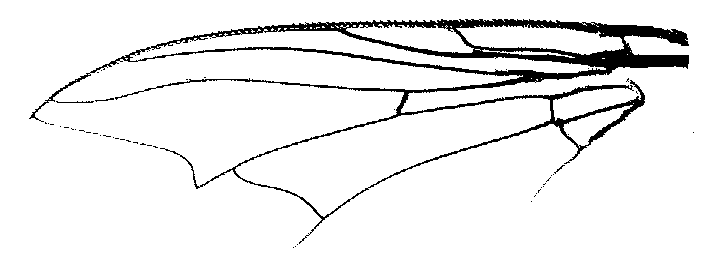

Supplement: Supplemental Information 4 [file peerj-cs-07-698-s004.zip › Supplemental File - Problem 1 Code and Raw Data/binary_images/B.peregrina.2.M.png]

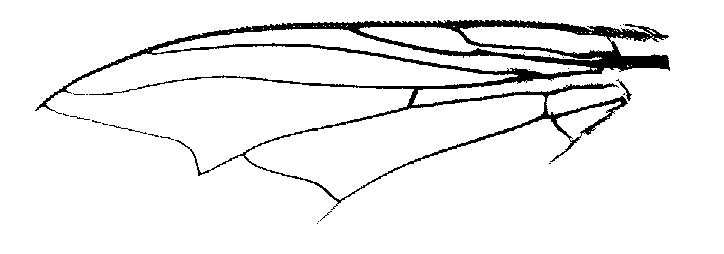

Supplement: Supplemental Information 4 [file peerj-cs-07-698-s004.zip › Supplemental File - Problem 1 Code and Raw Data/binary_images/B.peregrina.3.M.png]

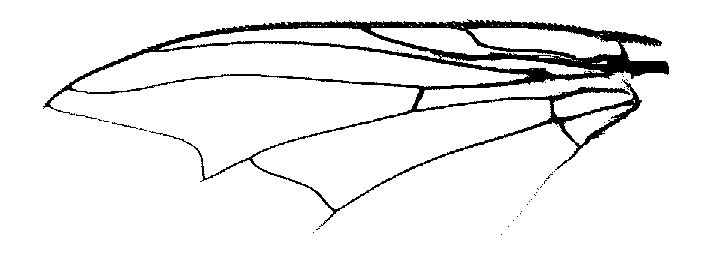

Supplement: Supplemental Information 4 [file peerj-cs-07-698-s004.zip › Supplemental File - Problem 1 Code and Raw Data/binary_images/B.peregrina.4.M.png]

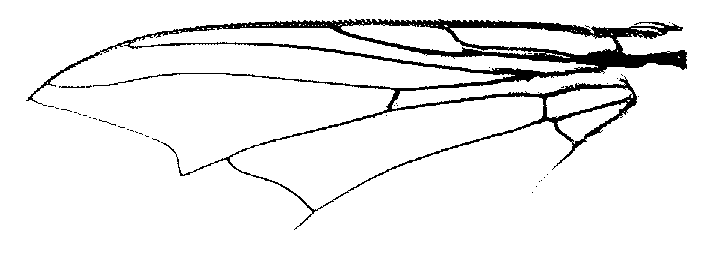

Supplement: Supplemental Information 4 [file peerj-cs-07-698-s004.zip › Supplemental File - Problem 1 Code and Raw Data/binary_images/B.peregrina.5.M.png]

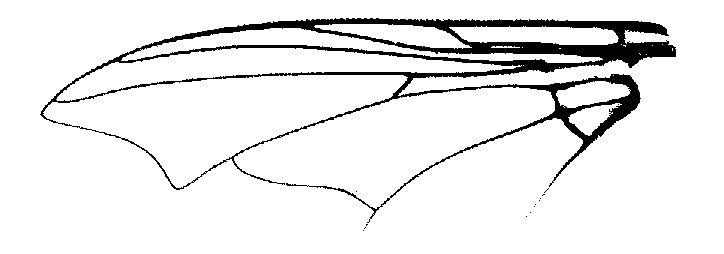

Supplement: Supplemental Information 4 [file peerj-cs-07-698-s004.zip › Supplemental File - Problem 1 Code and Raw Data/binary_images/C.megacephala.1.M.png]

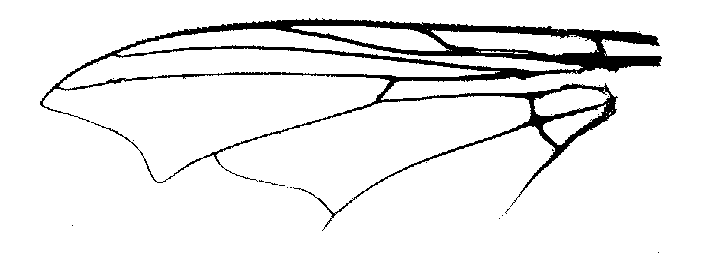

Supplement: Supplemental Information 4 [file peerj-cs-07-698-s004.zip › Supplemental File - Problem 1 Code and Raw Data/binary_images/C.megacephala.2.M.png]

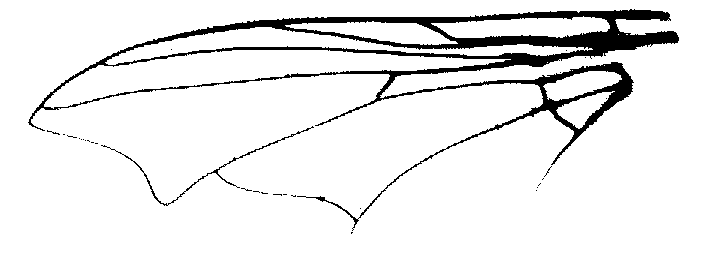

Supplement: Supplemental Information 4 [file peerj-cs-07-698-s004.zip › Supplemental File - Problem 1 Code and Raw Data/binary_images/C.megacephala.7.M.png]

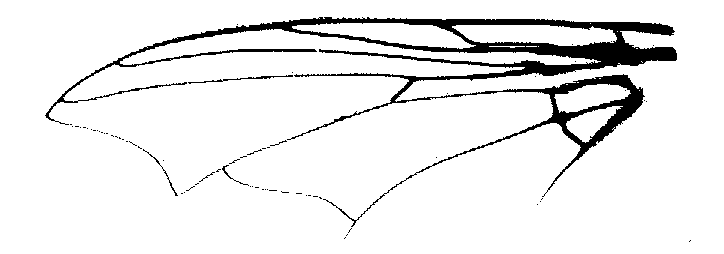

Supplement: Supplemental Information 4 [file peerj-cs-07-698-s004.zip › Supplemental File - Problem 1 Code and Raw Data/binary_images/C.megacephala.8.M.png]

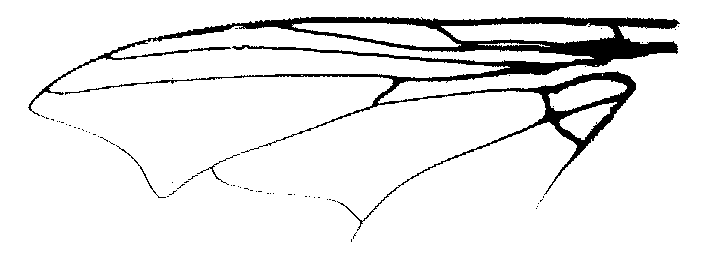

Supplement: Supplemental Information 4 [file peerj-cs-07-698-s004.zip › Supplemental File - Problem 1 Code and Raw Data/binary_images/C.megacephala.9.M.png]

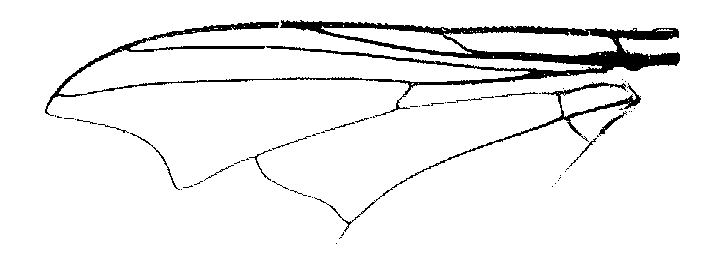

Supplement: Supplemental Information 4 [file peerj-cs-07-698-s004.zip › Supplemental File - Problem 1 Code and Raw Data/binary_images/C.nigripes.1.M.png]

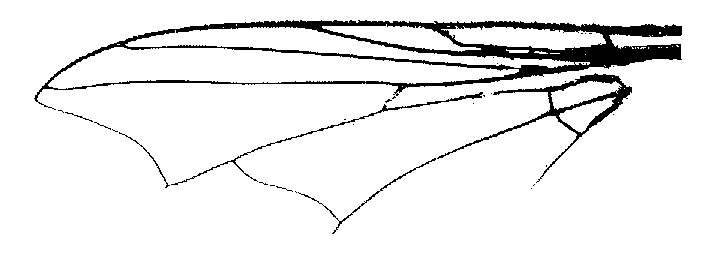

Supplement: Supplemental Information 4 [file peerj-cs-07-698-s004.zip › Supplemental File - Problem 1 Code and Raw Data/binary_images/C.nigripes.2.M.png]

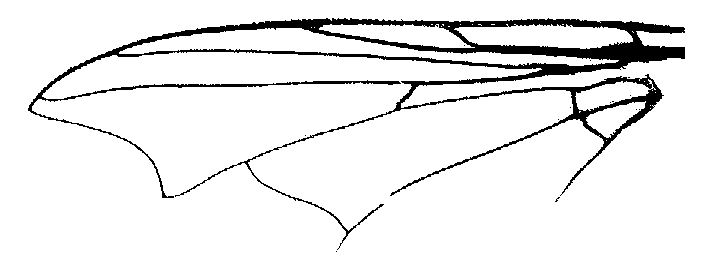

Supplement: Supplemental Information 4 [file peerj-cs-07-698-s004.zip › Supplemental File - Problem 1 Code and Raw Data/binary_images/C.nigripes.3.M.png]

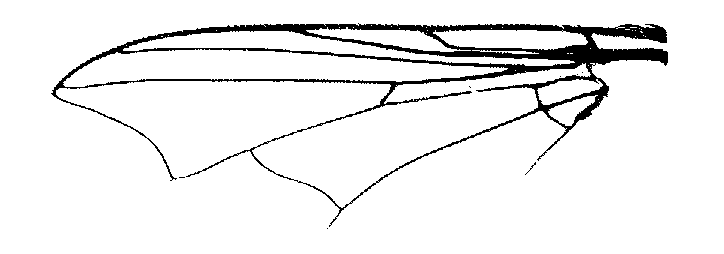

Supplement: Supplemental Information 4 [file peerj-cs-07-698-s004.zip › Supplemental File - Problem 1 Code and Raw Data/binary_images/C.nigripes.7.M.png]

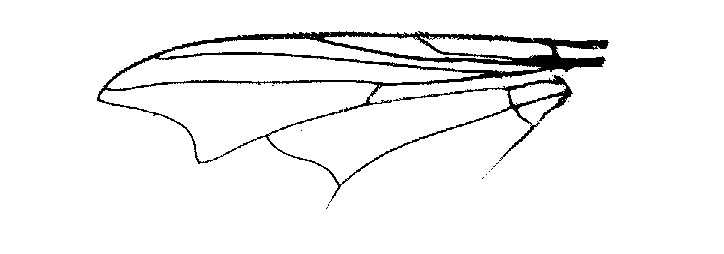

Supplement: Supplemental Information 4 [file peerj-cs-07-698-s004.zip › Supplemental File - Problem 1 Code and Raw Data/binary_images/C.nigripes.9.M.png]

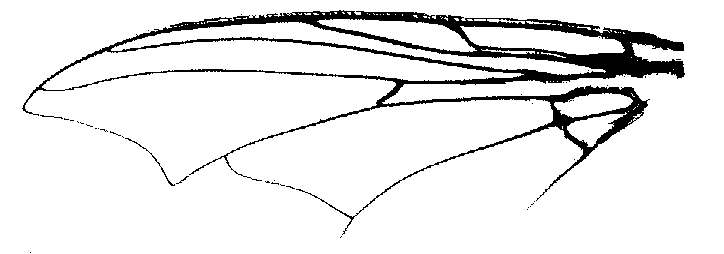

Supplement: Supplemental Information 4 [file peerj-cs-07-698-s004.zip › Supplemental File - Problem 1 Code and Raw Data/binary_images/C.pinguis.3.M.png]

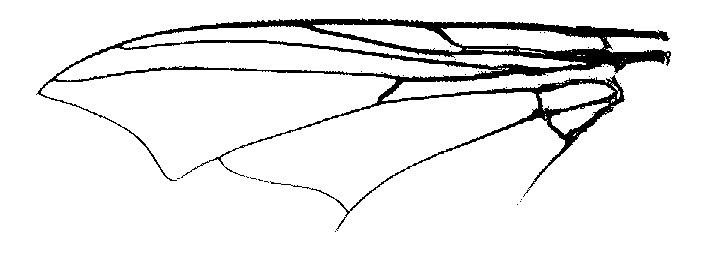

Supplement: Supplemental Information 4 [file peerj-cs-07-698-s004.zip › Supplemental File - Problem 1 Code and Raw Data/binary_images/C.pinguis.5.M.png]

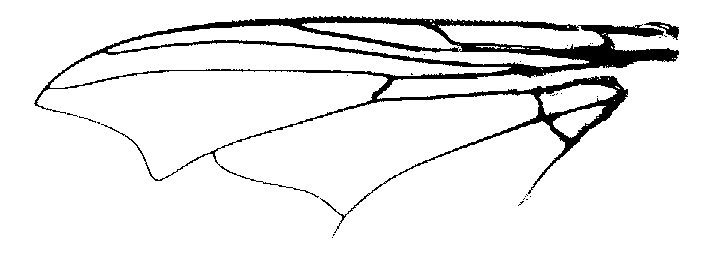

Supplement: Supplemental Information 4 [file peerj-cs-07-698-s004.zip › Supplemental File - Problem 1 Code and Raw Data/binary_images/C.pinguis.6.M.png]

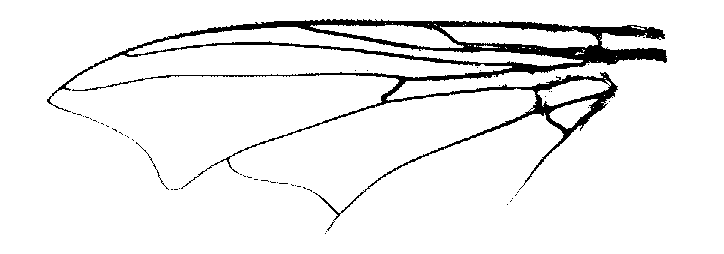

Supplement: Supplemental Information 4 [file peerj-cs-07-698-s004.zip › Supplemental File - Problem 1 Code and Raw Data/binary_images/C.pinguis.7.M.png]

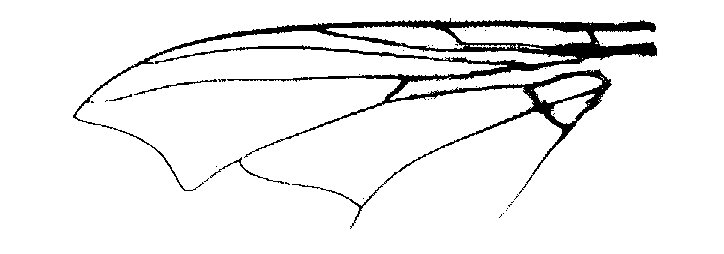

Supplement: Supplemental Information 4 [file peerj-cs-07-698-s004.zip › Supplemental File - Problem 1 Code and Raw Data/binary_images/C.pinguis.8.M.png]

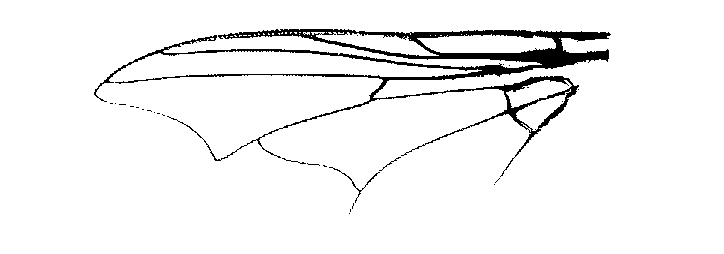

Supplement: Supplemental Information 4 [file peerj-cs-07-698-s004.zip › Supplemental File - Problem 1 Code and Raw Data/binary_images/C.rufifacies.1.M.png]

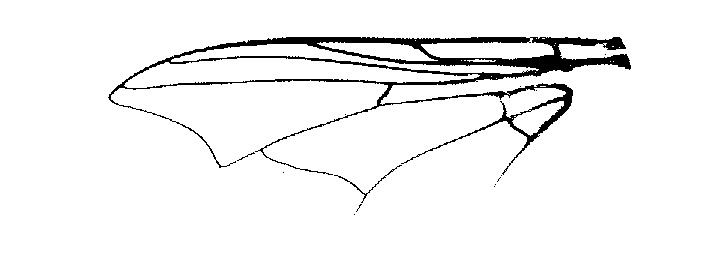

Supplement: Supplemental Information 4 [file peerj-cs-07-698-s004.zip › Supplemental File - Problem 1 Code and Raw Data/binary_images/C.rufifacies.2.M.png]

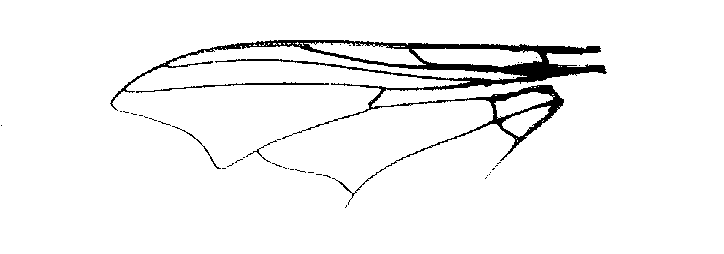

Supplement: Supplemental Information 4 [file peerj-cs-07-698-s004.zip › Supplemental File - Problem 1 Code and Raw Data/binary_images/C.rufifacies.3.M.png]

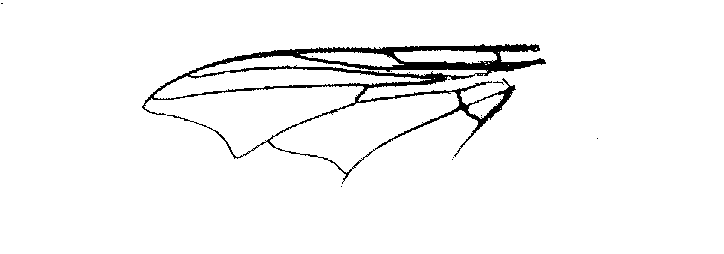

Supplement: Supplemental Information 4 [file peerj-cs-07-698-s004.zip › Supplemental File - Problem 1 Code and Raw Data/binary_images/C.rufifacies.4.M.png]

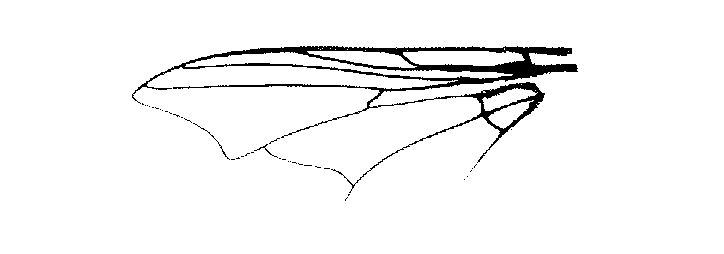

Supplement: Supplemental Information 4 [file peerj-cs-07-698-s004.zip › Supplemental File - Problem 1 Code and Raw Data/binary_images/C.rufifacies.5.M.png]

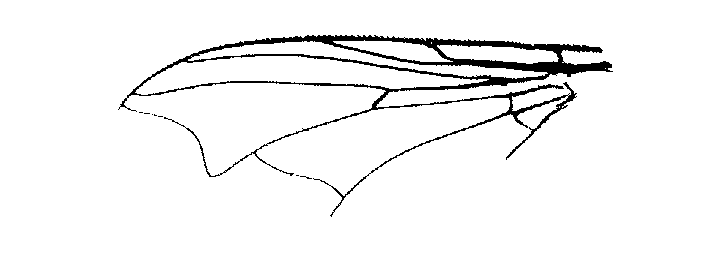

Supplement: Supplemental Information 4 [file peerj-cs-07-698-s004.zip › Supplemental File - Problem 1 Code and Raw Data/binary_images/C.villineuvi.1.M.png]

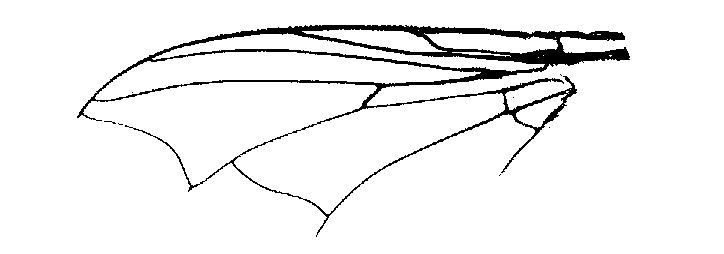

Supplement: Supplemental Information 4 [file peerj-cs-07-698-s004.zip › Supplemental File - Problem 1 Code and Raw Data/binary_images/C.villineuvi.2.M.png]

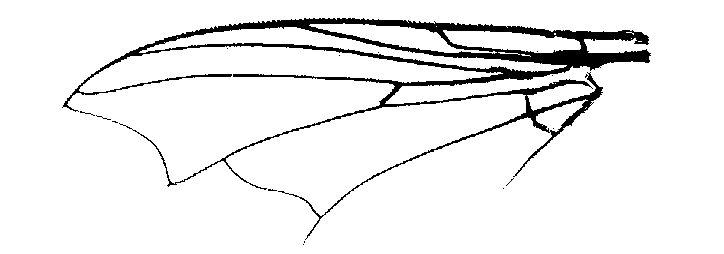

Supplement: Supplemental Information 4 [file peerj-cs-07-698-s004.zip › Supplemental File - Problem 1 Code and Raw Data/binary_images/C.villineuvi.3.M.png]

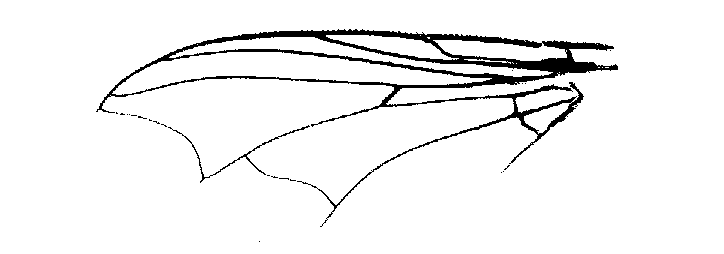

Supplement: Supplemental Information 4 [file peerj-cs-07-698-s004.zip › Supplemental File - Problem 1 Code and Raw Data/binary_images/C.villineuvi.4.M.png]

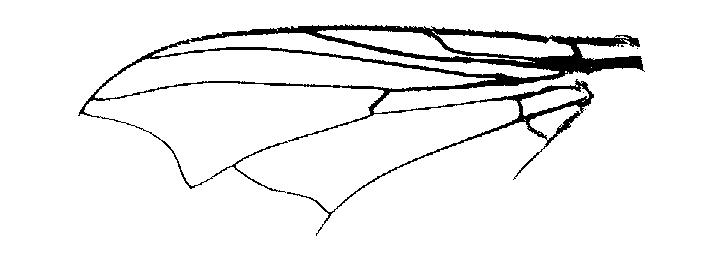

Supplement: Supplemental Information 4 [file peerj-cs-07-698-s004.zip › Supplemental File - Problem 1 Code and Raw Data/binary_images/C.villineuvi.5.M.png]

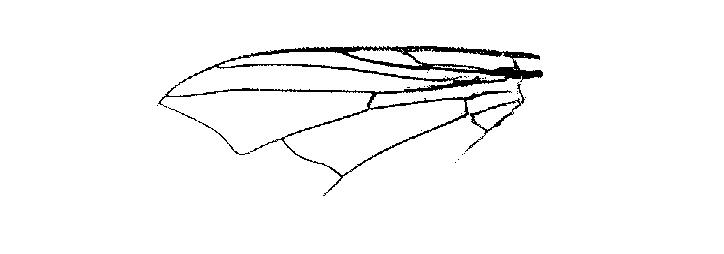

Supplement: Supplemental Information 4 [file peerj-cs-07-698-s004.zip › Supplemental File - Problem 1 Code and Raw Data/binary_images/L.cuprina.1.M.png]

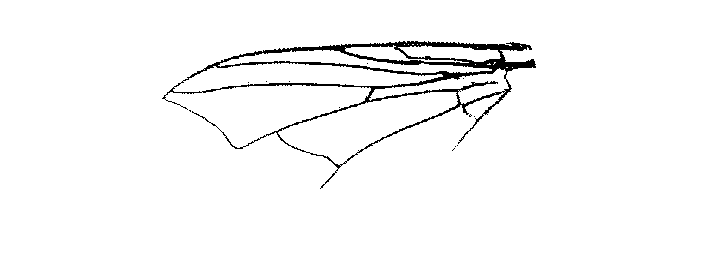

Supplement: Supplemental Information 4 [file peerj-cs-07-698-s004.zip › Supplemental File - Problem 1 Code and Raw Data/binary_images/L.cuprina.2.M.png]

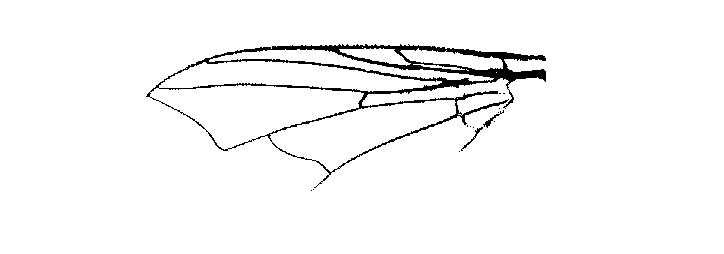

Supplement: Supplemental Information 4 [file peerj-cs-07-698-s004.zip › Supplemental File - Problem 1 Code and Raw Data/binary_images/L.cuprina.3.M.png]

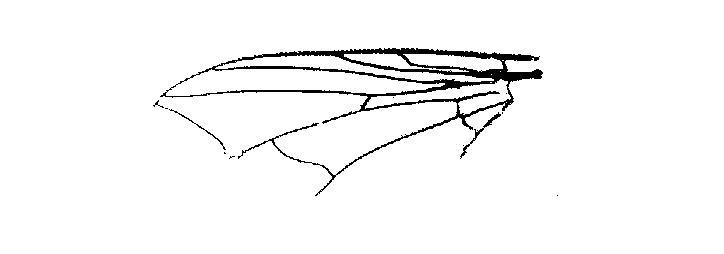

Supplement: Supplemental Information 4 [file peerj-cs-07-698-s004.zip › Supplemental File - Problem 1 Code and Raw Data/binary_images/L.cuprina.4.M.png]

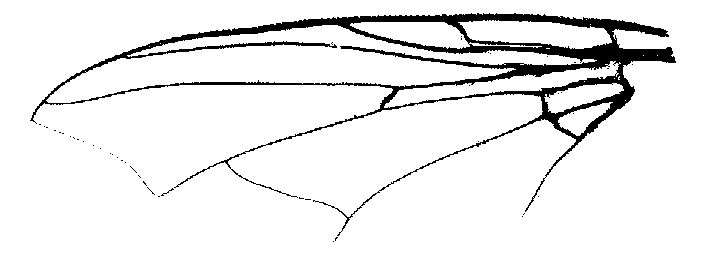

Supplement: Supplemental Information 4 [file peerj-cs-07-698-s004.zip › Supplemental File - Problem 1 Code and Raw Data/binary_images/L.porphyrina.1.M.png]

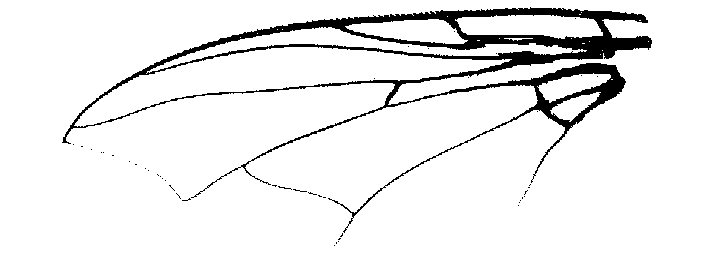

Supplement: Supplemental Information 4 [file peerj-cs-07-698-s004.zip › Supplemental File - Problem 1 Code and Raw Data/binary_images/L.porphyrina.10.M.png]

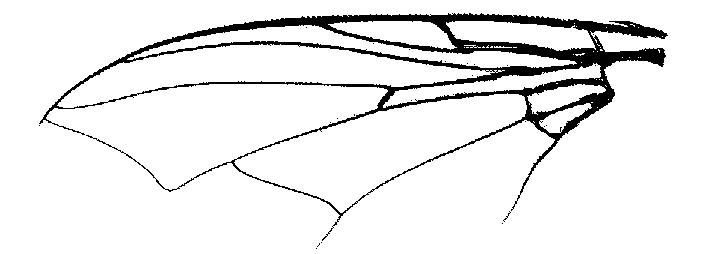

Supplement: Supplemental Information 4 [file peerj-cs-07-698-s004.zip › Supplemental File - Problem 1 Code and Raw Data/binary_images/L.porphyrina.4.M.png]

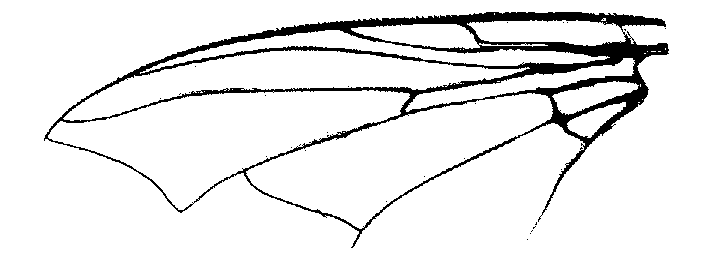

Supplement: Supplemental Information 4 [file peerj-cs-07-698-s004.zip › Supplemental File - Problem 1 Code and Raw Data/binary_images/L.porphyrina.5.M.png]

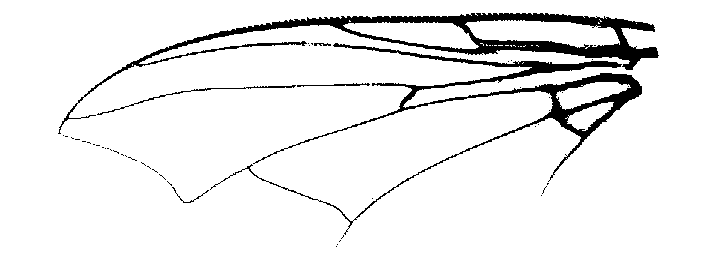

Supplement: Supplemental Information 4 [file peerj-cs-07-698-s004.zip › Supplemental File - Problem 1 Code and Raw Data/binary_images/L.porphyrina.9.M.png]

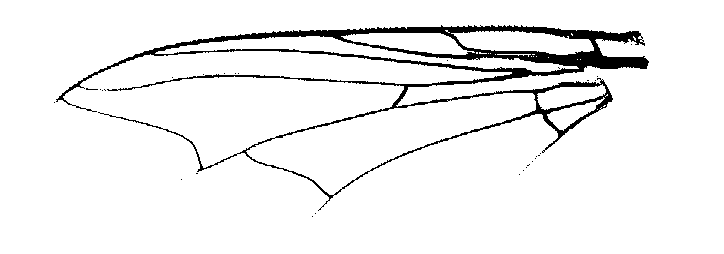

Supplement: Supplemental Information 4 [file peerj-cs-07-698-s004.zip › Supplemental File - Problem 1 Code and Raw Data/binary_images/P.albiceps.1.M.png]

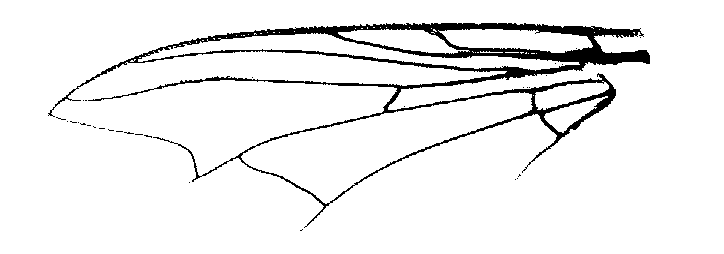

Supplement: Supplemental Information 4 [file peerj-cs-07-698-s004.zip › Supplemental File - Problem 1 Code and Raw Data/binary_images/P.albiceps.2.M.png]

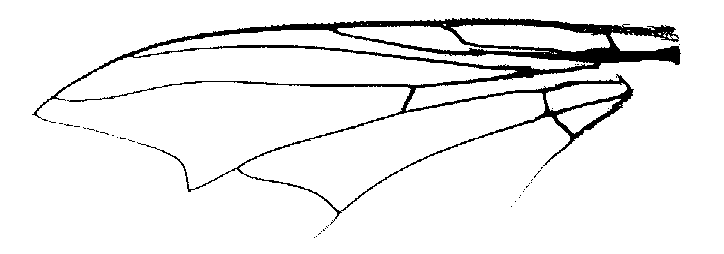

Supplement: Supplemental Information 4 [file peerj-cs-07-698-s004.zip › Supplemental File - Problem 1 Code and Raw Data/binary_images/P.albiceps.3.M.png]

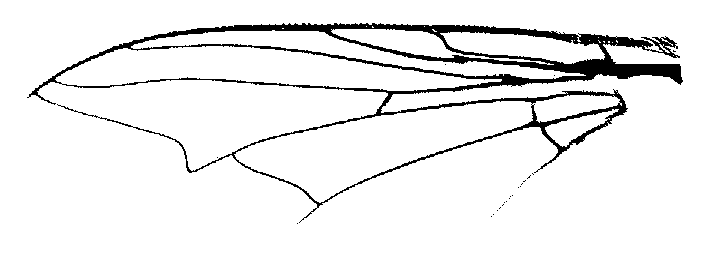

Supplement: Supplemental Information 4 [file peerj-cs-07-698-s004.zip › Supplemental File - Problem 1 Code and Raw Data/binary_images/P.albiceps.4.M.png]

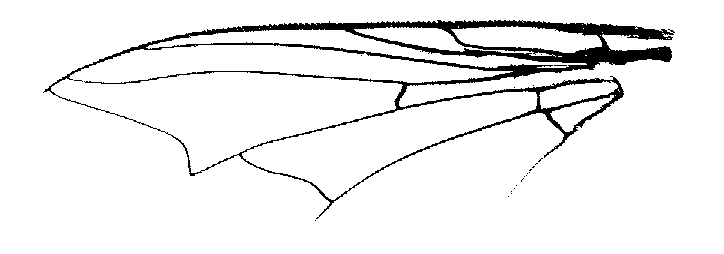

Supplement: Supplemental Information 4 [file peerj-cs-07-698-s004.zip › Supplemental File - Problem 1 Code and Raw Data/binary_images/P.albiceps.5.M.png]

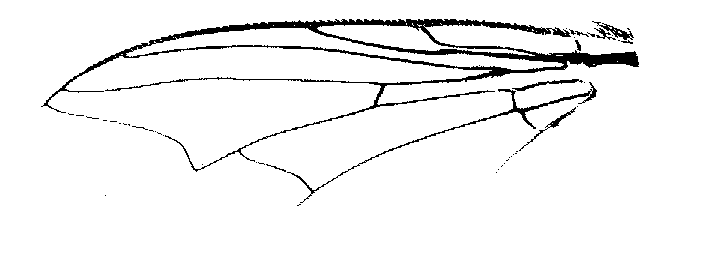

Supplement: Supplemental Information 4 [file peerj-cs-07-698-s004.zip › Supplemental File - Problem 1 Code and Raw Data/binary_images/P.misera.1.M.png]

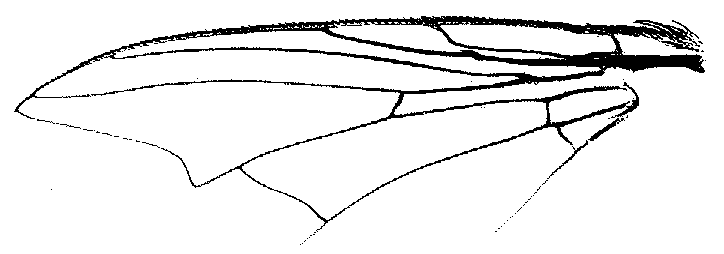

Supplement: Supplemental Information 4 [file peerj-cs-07-698-s004.zip › Supplemental File - Problem 1 Code and Raw Data/binary_images/P.misera.2.M.png]

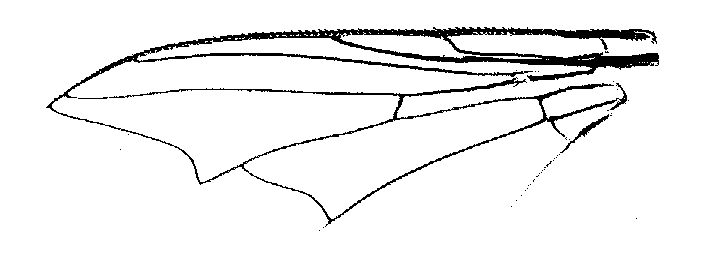

Supplement: Supplemental Information 4 [file peerj-cs-07-698-s004.zip › Supplemental File - Problem 1 Code and Raw Data/binary_images/P.misera.3.M.png]

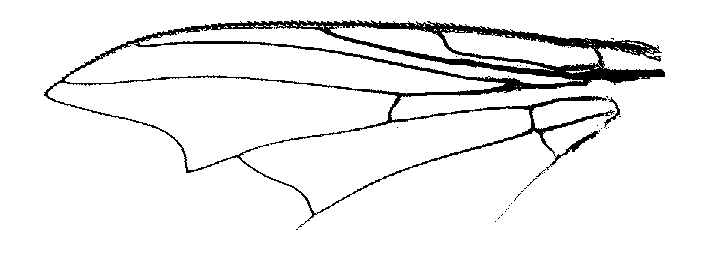

Supplement: Supplemental Information 4 [file peerj-cs-07-698-s004.zip › Supplemental File - Problem 1 Code and Raw Data/binary_images/P.misera.4.M.png]

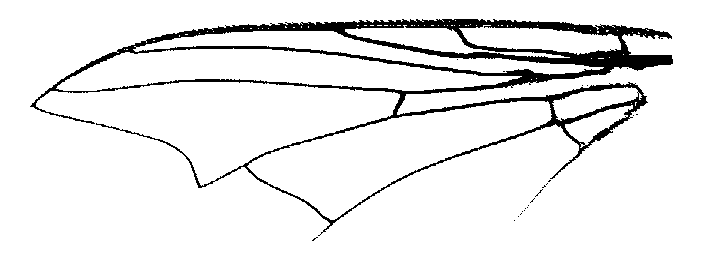

Supplement: Supplemental Information 4 [file peerj-cs-07-698-s004.zip › Supplemental File - Problem 1 Code and Raw Data/binary_images/P.misera.5.M.png]

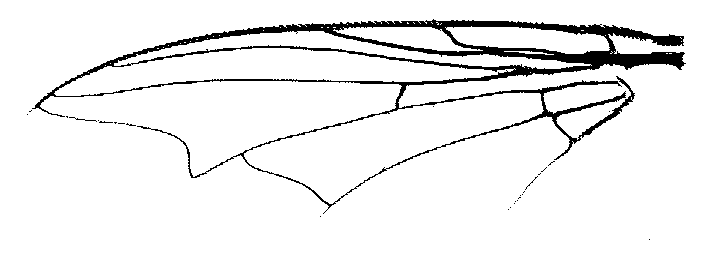

Supplement: Supplemental Information 4 [file peerj-cs-07-698-s004.zip › Supplemental File - Problem 1 Code and Raw Data/binary_images/S.dux.1.M.png]

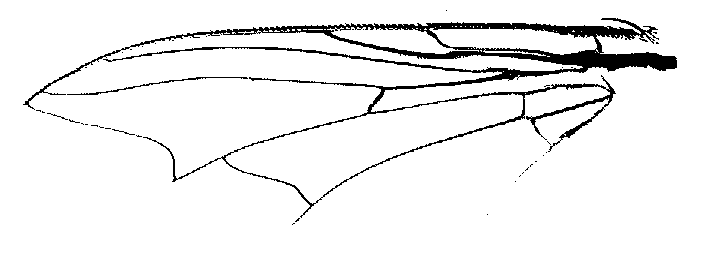

Supplement: Supplemental Information 4 [file peerj-cs-07-698-s004.zip › Supplemental File - Problem 1 Code and Raw Data/binary_images/S.dux.2.M.png]

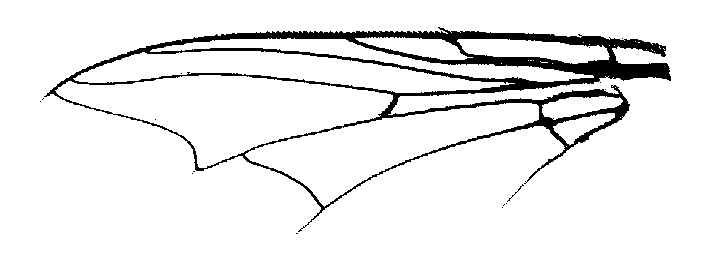

Supplement: Supplemental Information 4 [file peerj-cs-07-698-s004.zip › Supplemental File - Problem 1 Code and Raw Data/binary_images/S.dux.3.M.png]

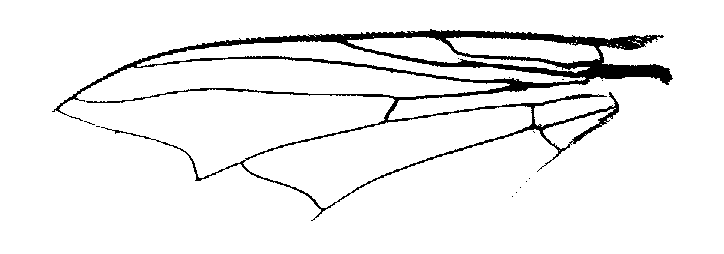

Supplement: Supplemental Information 4 [file peerj-cs-07-698-s004.zip › Supplemental File - Problem 1 Code and Raw Data/binary_images/S.dux.4.M.png]

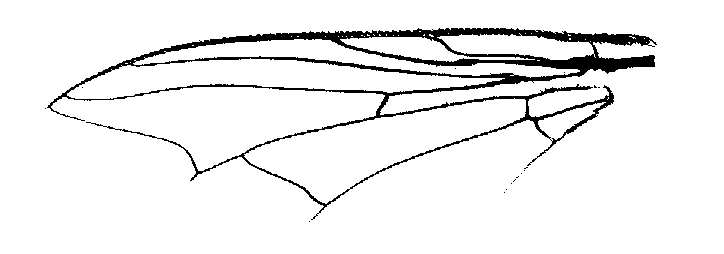

Supplement: Supplemental Information 4 [file peerj-cs-07-698-s004.zip › Supplemental File - Problem 1 Code and Raw Data/binary_images/S.dux.5.M.png]

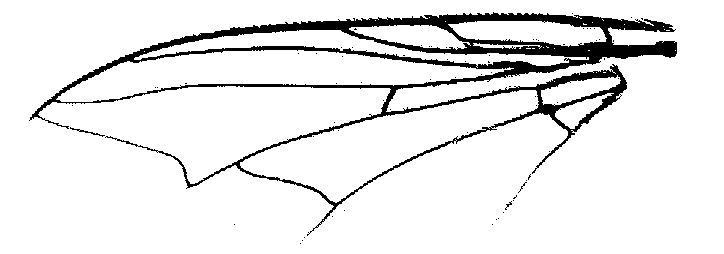

Supplement: Supplemental Information 4 [file peerj-cs-07-698-s004.zip › Supplemental File - Problem 1 Code and Raw Data/binary_images/S.princeps.1.M.png]

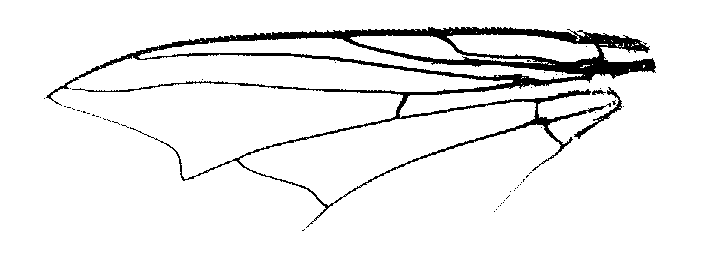

Supplement: Supplemental Information 4 [file peerj-cs-07-698-s004.zip › Supplemental File - Problem 1 Code and Raw Data/binary_images/S.princeps.2.M.png]

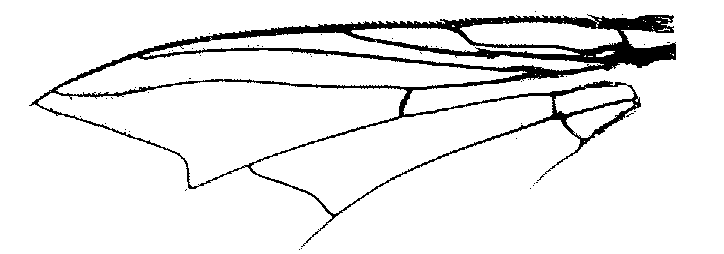

Supplement: Supplemental Information 4 [file peerj-cs-07-698-s004.zip › Supplemental File - Problem 1 Code and Raw Data/binary_images/S.princeps.3.M.png]

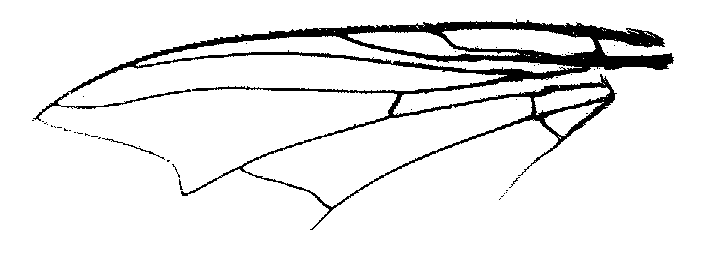

Supplement: Supplemental Information 4 [file peerj-cs-07-698-s004.zip › Supplemental File - Problem 1 Code and Raw Data/binary_images/S.princeps.4.M.png]

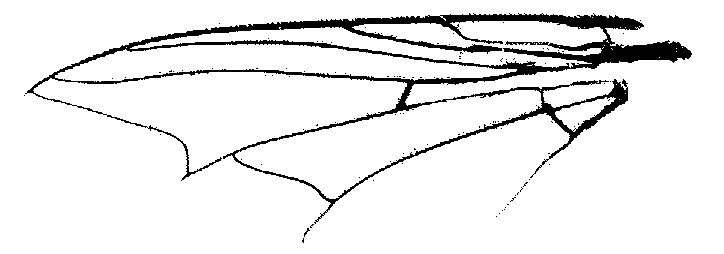

Supplement: Supplemental Information 4 [file peerj-cs-07-698-s004.zip › Supplemental File - Problem 1 Code and Raw Data/binary_images/S.princeps.5.M.png]

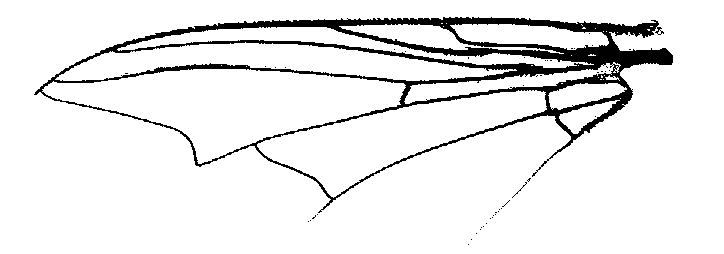

Supplement: Supplemental Information 4 [file peerj-cs-07-698-s004.zip › Supplemental File - Problem 1 Code and Raw Data/binary_images/S.ruficornis.1.M.png]

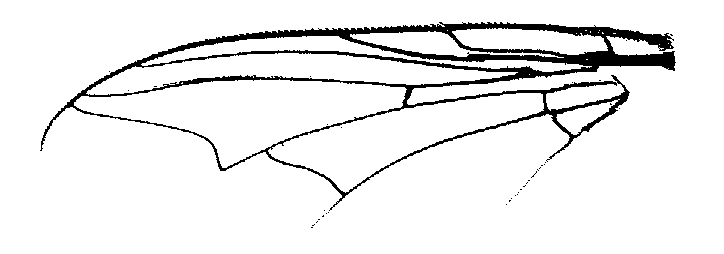

Supplement: Supplemental Information 4 [file peerj-cs-07-698-s004.zip › Supplemental File - Problem 1 Code and Raw Data/binary_images/S.ruficornis.2.M.png]

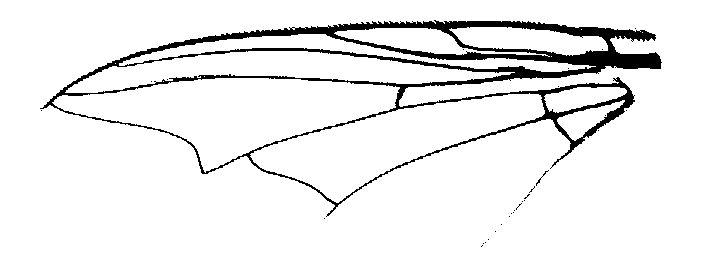

Supplement: Supplemental Information 4 [file peerj-cs-07-698-s004.zip › Supplemental File - Problem 1 Code and Raw Data/binary_images/S.ruficornis.3.M.png]

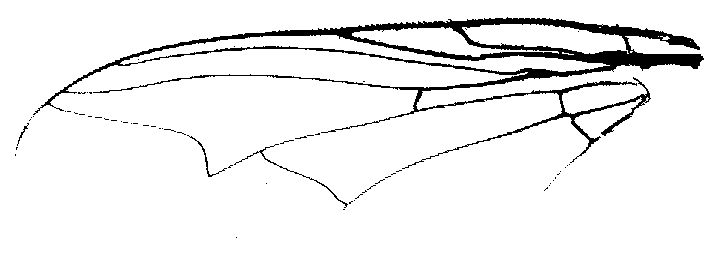

Supplement: Supplemental Information 4 [file peerj-cs-07-698-s004.zip › Supplemental File - Problem 1 Code and Raw Data/binary_images/S.ruficornis.4.M.png]

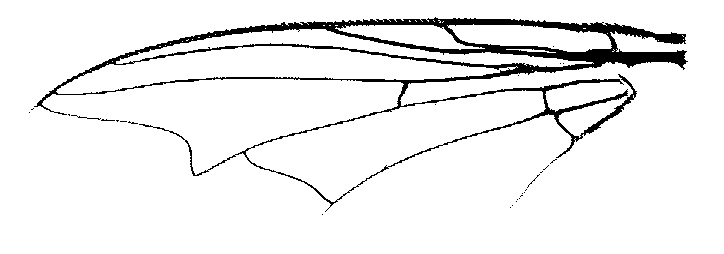

Supplement: Supplemental Information 4 [file peerj-cs-07-698-s004.zip › Supplemental File - Problem 1 Code and Raw Data/binary_images/S.ruficornis.5.M.png]
